# Comparative Transcriptome Profiling of Resistant and Susceptible Sugarcane Cultivars in Response to Infection by *Xanthomonas albilineans*

**DOI:** 10.3390/ijms20246138

**Published:** 2019-12-05

**Authors:** Mbuya Sylvain Ntambo, Jian-Yu Meng, Philippe C. Rott, Robert J. Henry, Hui-Li Zhang, San-Ji Gao

**Affiliations:** 1 National Engineering Research Center for Sugarcane, Fujian Agriculture and Forestry University, Fuzhou 350002, China; ntambos@africau.edu (M.S.N.); huilizhang2014@163.com (H.-L.Z.); 2 BGPI, INRA, CIRAD, SupAgro, Univ Montpellier, 34398 Montpellier, France; philippe.rott@cirad.fr; 3 Centre for Crop Science, Queensland Alliance for Agriculture and Food Innovation, University of Queensland, Brisbane, QLD 4072, Australia; robert.henry@uq.edu.au

**Keywords:** leaf scald, *Saccharum* spp., *Xanthomonas albilineans*, transcriptome analysis, disease resistance

## Abstract

Sugarcane (*Saccharum* spp. hybrids) is a major source of sugar and renewable bioenergy crop worldwide and suffers serious yield losses due to many pathogen infections. Leaf scald caused by *Xanthomonas albilineans* is a major bacterial disease of sugarcane in most sugarcane-planting countries. The molecular mechanisms of resistance to leaf scald in this plant are, however, still unclear. We performed a comparative transcriptome analysis between resistant (LCP 85-384) and susceptible (ROC20) sugarcane cultivars infected by *X. albilineans* using the RNA-seq platform. 24 cDNA libraries were generated with RNA isolated at four time points (0, 24, 48, and 72 h post inoculation) from the two cultivars with three biological replicates. A total of 105,783 differentially expressed genes (DEGs) were identified in both cultivars and the most upregulated and downregulated DEGs were annotated for the processes of the metabolic and single-organism categories, respectively. Kyoto Encyclopedia of Genes and Genomes (KEGG) pathway analysis of the 7612 DEGs showed that plant–pathogen interaction, spliceosome, glutathione metabolism, protein processing in endoplasmic reticulum, and plant hormone signal transduction contributed to sugarcane’s response to *X. albilineans* infection. Subsequently, relative expression levels of ten DEGs determined by quantitative reverse transcription-PCR (qRT-PCR), in addition to RNA-Seq data, indicated that different plant hormone (auxin and ethylene) signal transduction pathways play essential roles in sugarcane infected by *X. albilineans.* In conclusion, our results provide, for the first time, valuable information regarding the transcriptome changes in sugarcane in response to infection by *X. albilineans*, which contribute to the understanding of the molecular mechanisms underlying the interactions between sugarcane and this pathogen and provide important clues for further characterization of leaf scald resistance in sugarcane.

## 1. Introduction

Sugarcane (*Saccharum* spp. hybrids) is a major source of sugar and biofuel production worldwide, and it is one of the most valuable cash crops [1]. In addition to sugar and biofuel production, sugarcane is also used to produce fodder for livestock and cellulosic ethanol, a second-generation biofuel using by-products from cane sugar processing such as straw and cane fibers [2]. The current commercial cultivars are interspecific hybrids derived from crosses between a few parents belonging to *S. officinarum* (2*n* = 80, the noble sugarcane), *S. barberi* (2*n* = 111–120, the Indian sugarcanes), *S. sinense* (2*n* = 81–124, the Chinese sugarcanes), and the two wild species *S. spontaneum* (x = 8, 2*n* = 36–128) and *S. robustum* (x = 10, 2*n* = 60–80) [1,3]. These crosses resulted in highly polyploid and aneuploid cultivars with 2*n* = 100–130 chromosomes, thus hindering the study of genes which regulate most cellular processes in sugarcane [4,5].

Plant pathogens such as fungi, viruses, and bacteria are major constraints to sugarcane growth and productivity [6]. Unlike abiotic stresses, microbes causing biotic stresses are complex living entities that have the potential to penetrate and thrive inside the plant. Among these, the bacterium *Xanthomonas albilineans* causes a major sugarcane disease called leaf scald [7]. This disease results in significant reductions in sugarcane yield and juice quality in susceptible cultivars, preventing their commercial use [8,9]. Susceptible cultivars exhibit characteristic symptoms such as white, narrow, and sharply defined leaf stripes and ultimately complete necrosis and wilting of foliage, resulting in plant death [9,10,11]. Despite the importance of the disease, little is known about the molecular processes underlying the sugarcane defense mechanisms in response to *X. albilineans* infection.

Next-generation sequencing (NGS), and RNA-sequencing (RNA-seq) in particular, has become a popular and comprehensive technique to monitor transcriptional changes, providing a far more precise and high-throughput measurement of levels of transcripts and their isoforms as compared to hybridization or sequence-based approaches [12,13]. RNA sequencing technologies are increasing our understanding of the underlying genes and gene regulatory networks mediated by diverse biotic and abiotic factors [14,15]. Recently, this technology has been used to identify the molecular mechanisms triggering the response of sugarcane to infection by various pathogens. For example, the transcriptome analysis of susceptible sugarcane cultivars in response to *Sugarcane streak mosaic virus* and *Sorghum mosaic virus* have been investigated by Dong et al. (2017) [16] and Ling et al. (2018) [17], respectively; major genes and pathways that were triggered in sugarcane infected by pathogenic fungi, such as *Colletotrichum falcatu* causing red rot [18], *Sporisorium scitamineum* causing smut [19,20], and *Fusarium verticillioides* associated with pokkah boeng [21], have also been identified. Additionally, transcriptome analysis studies depicted the sugarcane response to infection by bacterial pathogens such as *Acidovorax avenae* subsp. *avenae* causing red stripe disease [22] and *Leifsonia xyli* subsp. *xyli* (*Lxx*) causing ratoon stunting disease [23,24]. These findings showed that many common genes and the pathways they are involved with constitute important clues for further investigation of sugarcane-pathogen interactions. These pathways include metabolism of carbohydrates, biosynthesis of secondary metabolites, plant–pathogen interaction, plant hormone signal transduction, and so on. Until now, the response of sugarcane to infection by *X. albilineans* has not been investigated using RNA-seq technology.

*Xanthomonas* is a large genus of *Gram* negative bacteria, collectively causing serious diseases in about 400 plant hosts that include a wide variety of economically important crops [25,26]. Various genes and pathways involved in host plant response to different *Xanthomonas* species using the RNA-seq technique have been identified. For example, in rice infected by *X. oryzae* pv. *oryzae* [27,28] and in wheat infected by *X. translucens* [29], genes involved in transcription factors (TFs) and different kinases, ethylene, jasmonic acid (JA), and secondary metabolites were found essential for resistance to these pathogens. In this study, we used the RNA-seq platform based on the Illumina NGS technology to investigate the molecular response to *X. albilineans* by comparing the transcriptome of two sugarcane cultivars, one resistant and one susceptible to leaf scald. Genes involved in resistance of sugarcane to this pathogen were identified. These candidate genes provide not only insights into the molecular mechanisms triggered in sugarcane by *X. albilineans* infection, but also constitute useful genetic resources for breeding sugarcane for *X. albilineans* resistance.

## 2. Results

### 2.1. Symptom Expression and Bacterial Population Size in Inoculated Sugarcane Leaves

No leaf scald symptoms were observed at 24 and 48 h post-inoculation (hpi), but pencil line streaks that are characteristic of leaf scald were observed at 72 hpi on both cultivars inoculated with *X. albilineans* (Figure S1A). A standard curve equation (Y = −3.3263x + 40.426, coefficient of determination (R^2^) = 0.9994, Efficiency = 99.82%) based on serial dilutions of pMD-albI plasmid was used for quantification of *X. albilineans* populations. Based on real-time quantitative PCR (qPCR) data, the population size of *X. albilineans* increased after inoculation and reached 890 and 250 copies of the genome of *X. albilineans* per reaction (copies/µL) at 72 hpi in the susceptible and the resistant cultivar, respectively (Figure S1B). Bacterial populations were therefore lower in the resistant than in the susceptible cultivar, thus confirming the difference in resistance between the two sugarcane cultivars.

### 2.2. RNA Sequencing and Assembly

The 24 libraries were sequenced using the Illumina sequencing platform and 57,563,635 to 62,175,579 raw reads were obtained for cultivar LCP 85-384 and 51,506,253 to 62,100,971 raw reads for cultivar ROC20 (Table S1). These raw reads were filtered with respect to quality (Q30), which resulted into 55,908,242 to 60,577,163 clean reads for LCP 85-384 and 50,221,927 to 60,390,747 clean reads for ROC20. A total of 614,270 transcripts were generated, with an average length of 736 bp and a N50 of 1045 bp. The total number of unigenes was 535,655 and they had an average length of 802 bp and a N50 of 1102 bp (Table 1). The size of most transcripts and unigenes ranged from 200–500 bp, accounting for 51% and 45% of total transcripts and unigenes, respectively. The longest sequence was 17,717 bp and the shortest sequence was 201 bp for both transcripts and unigenes databases (Table 1).

### 2.3. Gene Annotation of Assembled Transcripts

All unigenes were annotated by blasting their sequences against seven public databases. Significant annotation matches were found for 263,129 unigenes (49%) in the Nr database, 356,538 (67%) unigenes in the Nt database, 71,456 (13%) unigenes in the Kyoto Encyclopedia of Genes and Genomes (KEGG) Ortholog (KO) database, 147,109 (27%) unigenes in the Swiss-Prot database, 172,132 unigenes (32%) in the Protein family (Pfam) database, 199,233 unigenes (37%) in the Gene Ontology (GO) database, and 42,942 unigenes (8%) in the Eukaryotic Orthologous Groups (KOG) database (Table S2). Annotation was achieved for 407,743 (76%) unigenes in at least one of the seven databases. A total of 23,075 unigenes (4%) were present in all screened databases. Based on Nr annotations and E-value distribution, 46.1% of the mapped unigenes had an E-value < 1.0 × 10^−45^, while 21.4% of the matched unigenes showed very high homology with plant nucleotide sequences with an E-value lower than 1.0 × 10^−100^ (Figure S2). More than half (55.4%) of the sequences had a similarity with other plant sequences higher than 80% and only 11.2% had a similarity value lower than 60% (Figure S2). Most of the annotated sequences corresponded to known nucleotide sequences of other plant species with 48.9%, 24.5%, 10.7%, 9.6%, 3.9%, and 2.4%, matching with *Sorghum bicolor*, *Zea mays*, other species, *Setaria italica*, *Oryza sativa*, and *Saccharum* hybrid, respectively (Figure S2). These data support the synteny between sugarcane and other grasses, such as sorghum and maize.

### 2.4. Differential Expression Analysis of Assembled Transcripts

**Figure 1:**
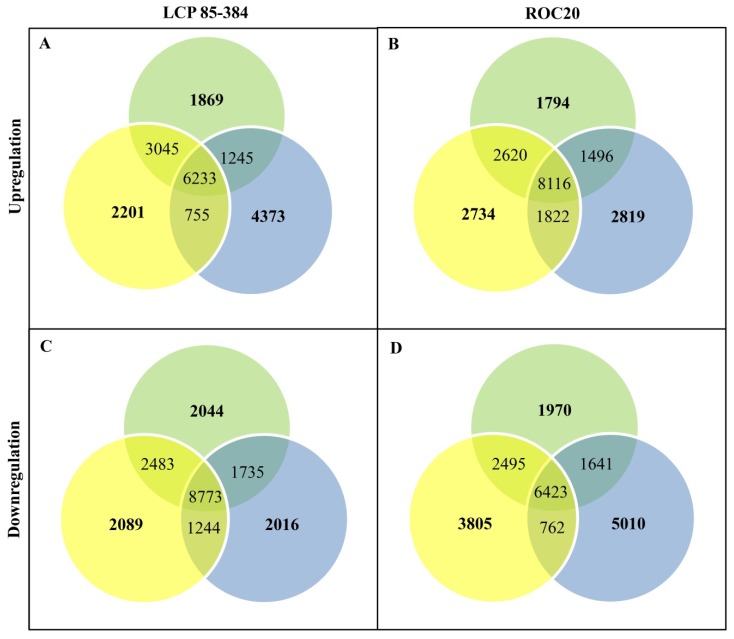
Venn diagrams of differentially expressed genes (DEGs) (Statistically significant ≥ 2-fold, *p*-value < 0.005). (**A**) and (**B**) Upregulated genes in cultivars LCP 85-384 and ROC20, respectively; and (**C**) and (**D**) downregulated genes in cultivars LCP 85-384 and ROC20, respectively. The sum of numbers in each circle represents the total number of DEGs for a given condition; the overlapping section of two-three circles represents common DEGs between different conditions: 24 (in yellow), 48 (in green), and 72 (in blue) hours post inoculation of sugarcane with *X. albilineans.*

Genes with a false discovery rate (adjusted *p*-value  <  0.005) and a fold change (FC)  ≥  2 were considered differentially expressed. Compared to the control plants inoculated with sterile XAS medium (S0_CK and R0_CK), the differentially expressed genes (DEGs) were identified at different time points after inoculation of plants with *X. albilineans,* and for both cultivars (Figure 1). A total of 21,401 upregulated and 22,106 downregulated DEGs were found for susceptible cultivar ROC20 and a total of 19,721 upregulated and 20,384 downregulated DEGs in resistant cultivar LCP 85-384 at 24-, 48-, and 72-hpi. Among treatments, 2201, 1869, and 4373 DEGs were specifically upregulated at 24, 48, and 72 hpi in LCP 85-384, and 2734, 1794, and 2819 DEGs were upregulated for the same respective time periods in ROC20 (Figure 1). A total of 2089, 2044, and 2016 DEGs were downregulated at 24, 48, and 72 hpi in LCP 85-384, respectively, and 3805, 1970, and 5010 DEGs were downregulated for the same respective time periods in ROC20. During the 24–72 hpi period, the number of upregulated DEGs was higher than the number of downregulated DEGs in LCP 85-384 (8443 vs. 6149), whereas the number of upregulated DEGs was lower than the number of downregulated DEGs in ROC20 (7347 vs. 10,785).

### 2.5. Gene Ontology Functional Analysis of Differentially Expressed Genes During Infection of Sugarcane by X. Albilineans

**Figure 2:**
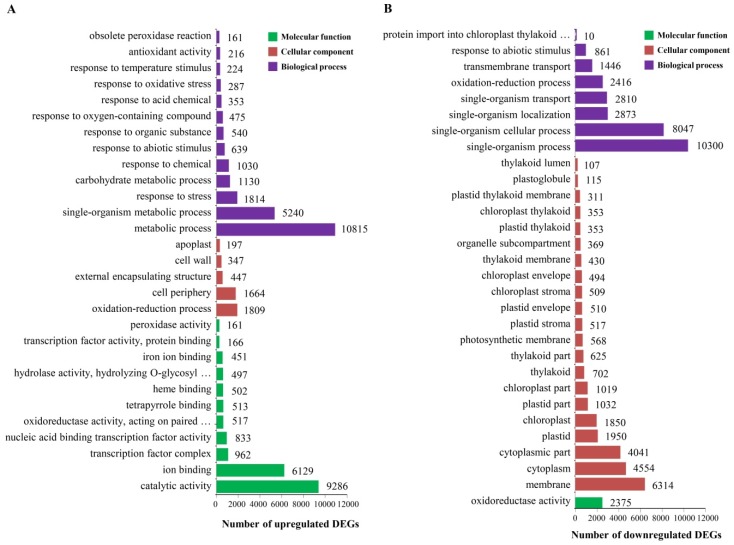
Assignment to Gene Ontology (GO) categories of DEGs of resistant sugarcane cultivar LCP 85-384 infected by *X. albilineans.* (**A**) Upregulated DEGs, (**B**) Downregulated DEGs.

GO terms for each of the unigenes were based on the best BLASTx hit from the Nr and Pfam databases using BlastGO software. Among the 424,963 upregulated and 379,130 downregulated transcripts of cultivar LCP 85-384, 47,922 upregulated and 57,861 downregulated DEGs, respectively, were assigned to one of three main GO categories: molecular function, cellular component, and biological process. The DEGs of LCP 85-384 were further distributed into 30 functional GO groups (Figure 2A,B). Of the upregulated DEGs, 22,924 were attributed to 13 groups of the biological process category and most of the genes (10,815) were involved in metabolic processes (Figure 2A). Another set of 4464 DEGs were assigned to the cellular component category and most of these genes (1809) were involved in an oxidation-reduction process, followed by 1644 genes involved in cell periphery. The molecular function category included 20,017 DEGs and, among those, 9286 were involved in catalytic activity, followed by 6129 genes involved in ion binding.

Of the downregulated DEGs of LCP 85-384, a total of 28,763 were assigned to the biological process category (Figure 2B). Most of them (10,300) were also involved in a single-organism process, followed by 8047 genes involved in a single-organism cellular process. A total of 26,623 DEGs were distributed in the cellular component category and most of them (14,909) in three GO groups: 6314 DEGs were in the membrane group, 4554 in the cytoplasm group, and 4041 DEGs in the cytoplasmic part group. The remaining 2375 DEGs were assigned to the molecular function category and all were included into the oxido-reductase GO group.

Among the 476,158 upregulated and 400,095 downregulated transcripts of cultivar ROC20, 49,013 upregulated and 62,742 downregulated DEGs, respectively, were assigned to one of the three main GO categories. The upregulated DEGs of ROC20 were further distributed into 29 functional GO groups whereas the downregulated DEGs were classified in 30 of these groups (Figure S3A,B). Of the upregulated DEGs, 43,034 were attributed to 19 groups of the biological process category and most of the genes (9750) were involved in metabolic processes (Figure S3A). Another set of 3548 DEGs were assigned to the cellular component category and most of these genes (1520) were related to cell periphery, followed by 941 genes involved in nuclear part. The molecular function category included 2431 DEGs and, among those, 746 were involved in transcription activity, followed by 461 genes involved in oxidoreductase activity.

Of the downregulated DEGs of ROC20, a total of 25,618 DEGs were assigned to the biological process category (Figure S3B). Most of them (10,350) were also involved in a single-organism process, followed by 6616 genes involved in a single-organism metabolic process. A total of 22,088 DEGs were distributed in the cellular component category and 8856 of them were found in two GO groups: the cytoplasm group (4675 DEGs) and the cytoplasmic part group (4181 DEGs). The remaining 15,036 DEGs were assigned to the molecular function category and most of them (11,640) were included into the catalytic activity GO group.

**Figure 3:**
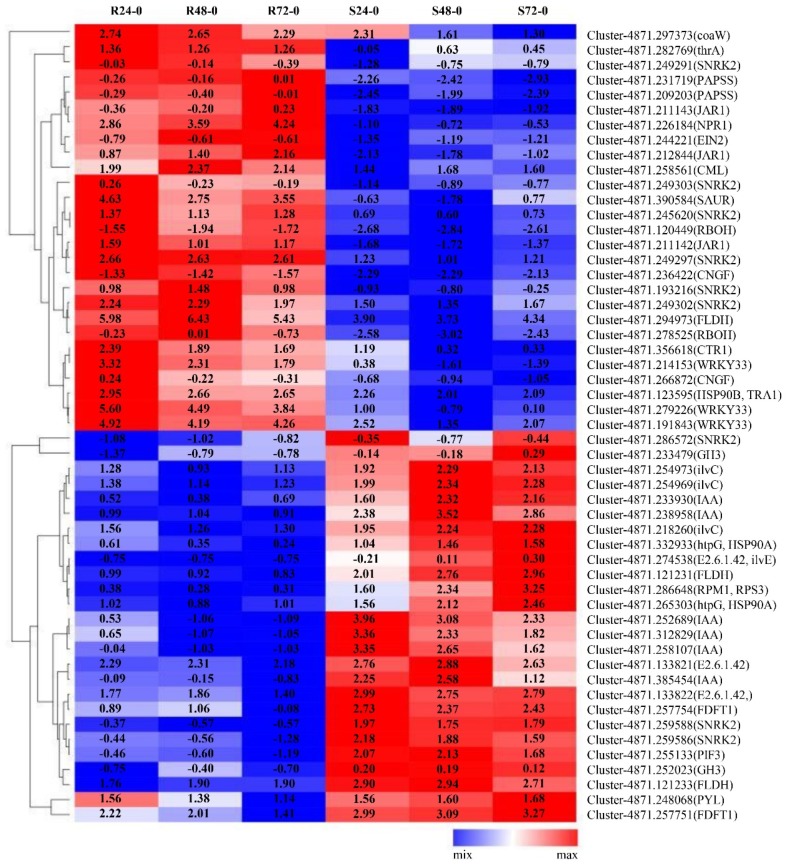
Hierarchical clustering of 53 DEGs of two sugarcane cultivars infected by *X. albilineans*. Each column represents a different experimental condition, S24_Xa vs. S0_CK (S24-0), S48_Xa vs. S0_CK (S48-0), S72_Xa vs. S0_CK (S72-0), R24_Xa vs. R0_CK (R24-0), R48_Xa vs. R0_CK (R48-0), and R72_Xa vs. R0_CK (R72-0). Colored background for each gene indicates the relative gene expression level that showed as log_10_ (fold change) (from high in red to low in blue).

Among the above mentioned DEGs, 53 DEGs were associated with plant disease-resistance genes (Table S3). A hierarchical clustering analysis of gene expression patterns revealed that 26 of the 53 DEGs were highly expressed in LCP 85-384 as compared with ROC20, whereas 27 DEGs were highly expressed only in ROC20 (Table S3 and Figure 3). The 26 upregulated DEGs in LCP 85-384 included some genes involved in pantothenate and CoA biosynthesis (coaW), sesquiterpenoid and triterpenoid biosynthesis (FLDH), monobactam biosynthesis (PAPSS and thrA), plant–pathogen interaction (CML, HSP90B, WRKY33, etc.), and plant hormone signal transduction (CTR1, EIN2, SAUR, SNRK2, etc.).

### 2.6. KEGG Enrichment Analysis of DEGs During Infection of Sugarcane by X. Albilineans

**Figure 4:**
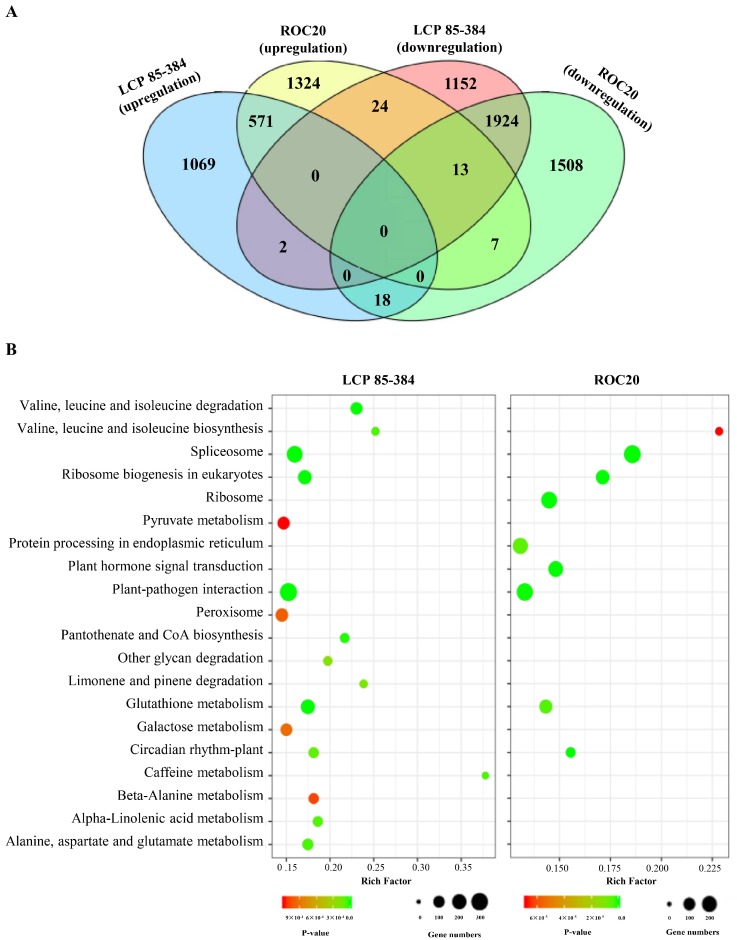
KEGG pathway classification and functional enrichment of DEGs (Statistically significant ≥ 2-fold, *p*-value < 0.005). (**A**) Venn diagrams of DEGs assigned to KEGG pathways in two sugarcane cultivars infected by *X. albilineans*. The upregulated DEGs of LCP 85-384 and ROC20 are shown in blue and yellow, respectively; the downregulated DEGs of LCP 85-384 24 and ROC20 are shown in pink and green, respectively. The overlapping section of two-four circles represents common DEGs. (**B**) Pathway functional enrichment of upregulated DEGs in LCP 83-384 (Left panel) and ROC20 (Right panel). The *X*-axis represents the enrichment factor (rich factor) which is the ratio of the foreground value (the number of DEGs) and the background value (total gene amount). The *Y*-axis shows the pathway names. A larger value of the rich factor indicates a higher enrichment value. The color indicates the *p*-value (high: red, low: green), A lower *p*-value refers to a more significant enrichment. Point size indicates DEG number and larger dots refer to higher numbers of DEGs.

To further study the biological pathways of DEGs triggered by *X. albilineans*, DEGs were annotated by blast analysis against the KEGG database using the KOBAS v. 2.0 software. A total of 7612 DEGs identified above were significantly enriched in 55 KEGG pathways. In response to *X. albilineans* infection, 3939 and 3728 DEGs significantly enriched in these KEGG pathways were active in resistant cultivar LCP 85-384 and susceptible cultivar ROC20, respectively (Figure 4A). When comparing the response of the two cultivars, more upregulated DEGs in LCP 85-384 (1861 vs. 1622) were activated in KEGG pathways, whereas more downregulated DEGs in ROC20 (2095 vs. 1078) were activated in KEGG pathways (Figure 4A). More than 100 upregulated DEGs were involved in each of the four KEGG pathways for both LCP 85-384 and ROC20: plant–pathogen interaction, spliceosome, ribosome biogenesis in eukaryotes, and glutathione metabolism (Figure 4B). KEGG pathways associated only with upregulated DEGs in LCP 85-384 included metabolism or degradation of several amino acids (alanine, aspartate, valine, etc.). KEGG pathways associated only with upregulated DEGs in ROC20 included ribosome, protein processing in endoplasmic reticulum, and plant hormone signal transduction.

More than 100 downregulated DEGs were involved in protein processing in endoplasmic reticulum for both LCP 85-384 and ROC20 (Figure S4). More than 100 downregulated DEGs were also involved in propanoate metabolism, pyrimidine metabolism, and ubiquitin mediated proteolysis in LCP 85-384, and in plant–pathogen interaction, purine metabolism, endocytosis, glutathione metabolism, aminoacyl-tRNA biosynthesis, and ribosome in ROC20. No KEGG pathway was associated with downregulated DEGs only in cultivar LCP 85-384, but 25 pathways were activated by downregulated DEGs only in ROC20 (endocytosis, mRNA surveillance pathway, oxidative phosphorylation, peroxisome, plant–pathogen interaction, purine metabolism, etc.).

### 2.7. Transcriptional Expression of Ten Genes Involved in Different Plant Hormone Signal Transduction Pathways

Ten DEGs were selected for confirmation by quantitative reverse transcription-PCR (qRT-PCR), in addition to Illumina RNA-seq data. These candidate genes involved in different pathways of plant hormone signal transduction, such as auxin signaling transduction (IAA, GH3, and SAUR), gibberellin signaling transduction (PIF3), absicisc acid (PYL and SNRK2), ethylene signaling transduction (CTR1 and EIN2), jasmonic acid signal transduction (JAR1), and salicylic acid signaling transduction (NPR1) (Table 2; Figure S5). Differences between RNA-seq data with log_2_ (fold change) and normalized qRT-PCR data did not reach significant levels (*p* > 0.05) for relative expression of each of the ten candidate genes at each time-point using the paired comparison T-test. The values of Prob > |t| ranged from 0.1028 to 0.9625. Overall, RNA-seq data presented that the five genes encoding IAA, SAUR, PYL, SNRK2, and CTR1 were highly expressed, but the three genes encoding GH3, EIN2, and JAR1 were depressed in the two cultivars; the *PIF3* gene (Cluster-4871.255133) was downregulated in resistant cultivar LCP 85-384, but upregulated in susceptible cultivar ROC20. In contrast, the *SAUR* (Cluster-4871.390584) and *NPR1* (Cluster-4871.226184) genes were upregulated in LCP 85-384 but downregulated in ROC20 (Table 2).

**Figure 5:**
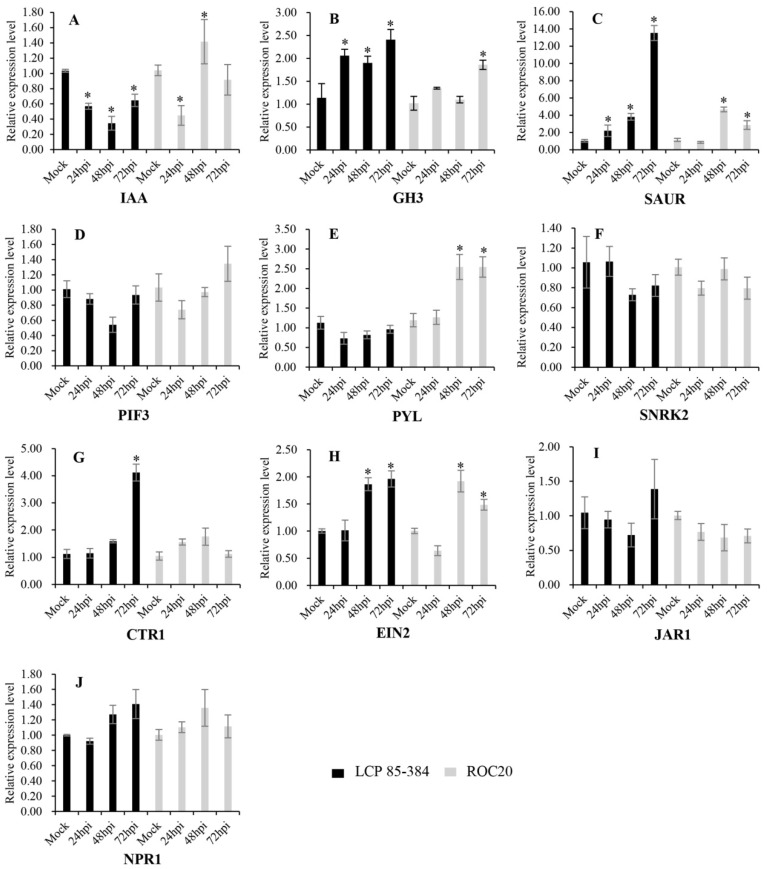
Expression analysis of ten DEGs (**A**–**J**) involved in different pathways based on quantitative reverse transcription-PCR (qRT-PCR) data. Each column represents the mean relative gene expression level at a given time point (mock = plants inoculated with sterile liquid medium XAS at 0 hpi). Vertical bars on top of each column represent the mean ± standard error for three biological replicates. Significant (*p* < 0.05) changes of relative expression level at each time point as compared to the mock are marked with an asterisk (*). (**A**) Auxin-responsive protein IAA (*IAA*, Cluster-4871.238958); (**B**) Auxin responsive GH3 gene family (*GH3*, Cluster-4871.233479); (**C**) SAUR family protein (*SAUR*, Cluster-4871.390584); (**D**) Phytochrome-interacting factor 3 (*PIF3*, Cluster-4871.255133); (**E**) Abscisic acid receptor PYR/PYL family (*PYL*, Cluster-4871.248068); (**F**) Serine/threonine-protein kinase SRK2 (*SNRK2*, Cluster-4871.245620); (**G**) K14510 Serine/threonine-protein kinase CTR1 (*CTR1*, Cluster-4871.356618); (**H**) Ethylene-insensitive protein 2 (*EIN2*, Cluster-4871.244221); (**I**) Jasmonic acid-amino synthetase (*JAR1*, Cluster-4871.211143); (**J**) Regulatory protein NPR1 (*NPR1*, Cluster-4871.226184).

On the other hand, significant changes (*p* < 0.05) of transcript expression levels were found for six of these ten DEGs in response to sugarcane infection by *X. albilineans* across the 24–74 hpi time period (Figure 5). These six genes encoded IAA, GH3, SAUR, PYL, CTR1, and EIN2. The *IAA* gene (Cluster-4871.238958) was significantly downregulated in LCP 85-384, but not in susceptible cultivar ROC20, except at 24 hpi. The *GH3* (Cluster-4871.233479) and *SAUR* genes were significantly upregulated in both cultivars, especially in LCP 85-384. The *PYL* gene was significantly upregulated in ROC20 at 48 and 72 hpi, but not in LCP83-384. The *CTR1* gene (Cluster-4871.356618) was significantly upregulated in LCP 85-384 at 72 hpi, but not in ROC20. The *EIN2* gene (Cluster-4871.244221) was significantly upregulated in both cultivars at 48 hpi and 72 hpi. However, three candidate genes (*PIF3*, *SNRK2*, and *JAR1*) associated with plant hormone signal transduction pathways were not significantly regulated in sugarcane in response to infection by *X. albilineans*.

## 3. Discussion

### 3.1. Global Patterns of Gene Transcription in Sugarcane in Response to Infection by X. Albilineans

In comparison to traditional Sanger sequencing that results in relatively long read lengths (700–100 bp) but a limited amount of data, NGS technologies provide millions of relatively short read length (30–500 bp) and gigabases of sequence data per run [30]. NGS followed by the use of sophisticated bioinformatics tools for data analysis, including high performance de novo transcriptome assembly, has recently emerged to facilitate analysis of the transcriptional changes during plant–pathogen interactions [13]. In this study, 24 sugarcane cDNA libraries were produced for the first time using the high-throughput RNA-seq technique to elucidate the molecular mechanisms triggered in sugarcane in response to infection by *X. albilineans*, the causal agent of leaf scald. Overall, the number of DEGs in LCP 85-384 (resistant cultivar) was lower than the number of these genes in ROC20 (susceptible cultivar), suggesting that the global gene expression within the first 72 h after sugarcane inoculation was less intense in the resistant cultivar than in the susceptible one. However, the number of upregulated DEGs was higher than the number of downregulated DEGs in LCP 85-384 and the number of upregulated DEGs was lower than the number of downregulated DEGs in ROC20. This indicated that gene upregulation was more active in the resistant cultivar than in the susceptible one. Conversely, gene downregulation appeared to be predominant in the susceptible cultivar rather than in the resistant one.

### 3.2. Major Gene Categories Involved in Response of Sugarcane to Infection by X. Albilineans

GO enrichment analysis revealed that the majority of upregulated DEGs (>5000) were attributed to categories related to metabolic process, catalytic activity, ion binding, and single-organism metabolic process of sugarcane during infection by *X. albilineans*. The metabolic process appeared to be a common biological process involved in the resistant (LCP 85-384) and the susceptible (ROC20) sugarcane cultivar after inoculation with the pathogen. Metabolic processes are organic processes (in a cell or organism) that are necessary for life and important features identified during infection of sugarcane by *S. scitamineum* [20] and in *Saccharum narenga* (Nees ex Steud.) in response to water deficit [31]. The majority of downregulated DEGs (>5000) were attributed to categories related to single-organism process, single-organism cellular process, single-organism metabolic process, catalytic activity, and membrane (cellular component) in response of the sugarcane to *X. albilineans* infection. Single-organism process was the most common biological process negatively involved during pathogen colonization, thus suggesting that this biological process was suppressed by *X. albilineans* to promote its spread within the host plant. Indeed, numerous DEGs related to single-organism metabolic process and catalytic activity were involved in up and downregulation of the sugarcane defense response against *X. albilineans*.

### 3.3. Major Enriched KEGG Pathways Associated with the Response of Sugarcane to Infection by X. Albilineans

KEGG analysis revealed that four important pathways (>200 DEGs for each pathway) related to glutathione metabolism, plant–pathogen interaction, ribosome biogenesis in eukaryotes, and spliceosome were upregulated in both the resistant and the susceptible sugarcane cultivar after infection by *X. albilineans*. On the other hand, glutathione metabolism and plant–pathogen interaction were also downregulated pathways at certain time points after inoculation with the pathogen. Glutathione (GSH) is a key factor for cellular redox homeostasis and tolerance against biotic and abiotic stresses [32,33]. Glutathione metabolism is required for *Arabidopsis* immunity during the hypersensitive response to fungi of the genus *Colletotrichum* [34]. Glutathione biosynthesis was enriched during the response of sugarcane to *A. avenae* subsp. *avenae* [22]. Several *glutathione-S-transferase* (GST) genes were induced in sugarcane post-inoculation by *S. sporisorium* [20,35], and particularly in a resistant sugarcane genotype, the GST activity was increased [35]. In this study, both upregulated and downregulated DEGs were involved in the Glutathione metabolism but upregulated DEGs were predominant, suggesting that this antioxidant peptide plays a key role in the defense response of sugarcane against *X. albilineans*. 

Plant–pathogen interaction is a multifaceted process, mediated by the pathogen- and plant-derived molecules and this process mostly includes proteins, sugars, and lipopolysaccharides [36]. This interaction involves two-way communication whereby the plant recognizes and defends itself against a potential pathogen, and the pathogen manipulates the biology of the plant to create a suitable environment for its growth and reproduction. Recognition of the pathogen or microbe-associated molecular patterns (PAMPs or MAMPs) is a first step of active plant defense known as PAMP-triggered immunity (PTI). Recognition by the plant of specific effectors produced by the pathogen is a second step of strong plant defense known as effector-triggered immunity (ETI). ETI involves localized programmed cell death during which the growth of the pathogen is specifically stopped [37,38]. On the other hand, the pathogen suppresses different components of PTI by effector proteins delivered into the cytoplasm of the host plant [36]. Resistant sugarcane cultivar LCP 85-384 used in this study activated more upregulated DEGs involved in plant–pathogen interaction pathways than susceptible cultivar ROC20 after inoculation with *X. albilineans*. Furthermore, only ROC20 had downregulated DEGs involved in these pathways, thus indicating that most identified genes encode proteins linked to disease resistance mechanisms. Numerous genes associated with disease resistance were identified in sugarcane during infection by *S. scitamineum*, such as signal transduction, defense proteins (pathogenesis-related and several disease-resistance proteins), hormone response, and secondary metabolites [20,39].

### 3.4. Plant Hormone Signal Transduction Pathways Involved in the Response of Sugarcane to Infection by X. Albilineans

Plant hormones play essential roles in the regulation of various physiological processes of plants, including development, growth, reproduction, and response to a wide range of biotic and abiotic stresses. Colonization of plants by pathogens results in level changes of various phytohormones [40]. Plant hormones such as JA, ethylene, and indole acetic acid (IAA) are involved in defense against pathogens [41,42]. Genes related to auxin, abscisic acid (ABA), ethylene, and JA signaling pathways have also been associated with infection of sugarcane by various pathogens [18,22,23]. To regulate plant growth and development, auxin can induce the expression of gene groups such as the Aux/IAA family, the GH3 family and the small auxin-up RNA (SAUR) family [43]. In this study, *IAA* showed downregulation in LCP 85-384, whereas *GH3* and *SAUR* showed upregulation responses in the two sugarcane cultivars. However, upregulation of these latter genes was higher in the resistant cultivar as compared to the susceptible one, thus suggesting their contribution to sugarcane resistance against *X. albilineans.* After whip emission during development of sugarcane smut, auxin was the hormone with the highest number of upregulated genes [44]. Sugarcane’s response to this disease included genes involved in auxin influx/efflux, auxin-amino acid hydrolase, and auxin responsive proteins such as Aux/IAA, SAUR, and auxin-induced β-glucosidase [44]. Additionally, genes related to the metabolism of auxin were all up-regulated in susceptible variety inoculated by *Lxx* mostly at 60 days [23] and similar hormonal changes were observed in sugarcane trigged by *Lxx* infection [45]. The auxin responsive *GH3* gene also plays important roles in plant defense responses in *Arabidopsis* and rice [41]. Overexpression of *GH3-8* resulted in enhanced resistance to the rice pathogen *Xanthomonas oryzae* pv. *oryzae* causing bacterial blight of rice [46]. This latter resistance is independent of salicylic acid (SA) and JA signaling [41].

The dual roles of ABA in plants in response to resistance tobiotrophic and necrotrophic pathogens depended on environmental conditions during experiments [47]. In the ABA signal transduction pathway, three core components were included; pyrabactin resistance 1 (PYR1) and PYR1-like (PYL)/regulatory components of ABA receptor (RCAR), protein phosphates 2C (PP2C), and sucrose non-fermenting 1-related protein kinase 2 (SNRK2) [48]. The PYLs interact with PP2Cs and prevent them from inhibiting SNRK2s, which stimulates stomatal closure by phosphorylating ion channels, and ABA-responsive gene expression by phosphorylating TFs (such as ABA-responsive element-binding factors, ABFs), and control other processes by phosphorylating other substrates [49,50]. In this study, we proposed that ABA signal transduction pathway may play negative or unimportant role on sugarcane resistance to *X. albilineans*, as validated by the upregulation of PYL (a receptor complex of ABA) in susceptible cultivar ROC20 but no significant change in the transcripts expression of SNRK2 gene in two cultivars. Recently, a similar result presented by Fu et al. (2019) showed that the susceptible genotype accumulated higher level ABA receptor PYR/PYL than the resistant genotype at 24 hpi under *Lxx* infection [24]. An additional case by Su et al. (2016) demonstrated that a protein phosphates 2C (PP2C, a negative regulator) that was responsible for ABA signaling was upregulated in smut-susceptible cultivar ROC22, but remained unchanged in smut-resistance cultivar Yacheng05-179, suggesting the ABA pathway was not involved or at least unimportant in the defense response of sugarcane to *S. scitamineum* [51].

The phytohormone ethylene also plays pivotal roles in plant response to developmental and environmental signals. In the ethylene signal transduction pathway, numerous key signaling components are involved, such as EIN2 (ethylene insensitive 2) and EIN3 (ethylene insensitive 3), ETR (ethylene response), and CTR1 (constitutive triple response 1) [52,53]. CTR1, a Raf-like MAPKKK (mitogenactivated protein kinase kinase kinase) family protein, has a role in regulating upstream of EIN2 and downstream of receptors (ETR) [54]. In our study, gene *CTR1* was significantly upregulated in resistant cultivar LCP 85-384, whereas it remained stable in susceptible ROC20, while *EIN2* was significantly upregulated in both sugarcane cultivars following infection by *X. albilineans.* This result suggested that the expression of *CTR1* and *EIN2* was essential during the resistant and susceptible responses of sugarcane against *X. albilineans*. Interestingly, the two proteins EIN3 (a plant-specific transcription factor) and ERF1 (ethylene response factor 1), acting downstream of EIN2 [55], were also upregulated in a sugarcane cultivar resistant to smut following infection by *S. scitamineum* [51]. The ethylene signal transduction pathway may therefore be involved in the regulation of plant defense responses against various pathogens.

## 4. Materials and Methods

### 4.1. Plant Growth and Inoculation with the Pathogen

Sugarcane cultivars LCP 85-384 (resistant to leaf scald) [56] and ROC20 (susceptible to leaf scald) [57], that originated from the Louisiana State University Agricultural Center, Sugar Research Station (St. Gabriel, LA, USA) and Taiwan Sugar Corporation (Taiwan, China), respectively, were used for inoculation with *X. albilineans*. Sugarcane plants of LCP 85-384 and ROC20 cultivars were grown in a growth chamber at 28 °C with 60% humidity and a 16/8 h light/dark photoperiod. A single colony of *X. albilineans* strain Xa-FJ1 was suspended in one mL XAS liquid medium and cultured at 28 °C for 48 h with constant shaking at 200 rpm. One μL of this latter culture was then added to 40 mL of fresh XAS liquid medium and cultured at 28 °C for about 10 h [58]. The bacterial suspension was diluted to 10^8^ CFU/mL for plant inoculation. At 3–5 leaf stage and when plants were approximately 15–20 cm high, 18 plants of each cultivar were inoculated with *X. albilineans* by cutting the leaf blades at mid-length with sterile scissors previously dipped in the bacterial suspension [58]. Six control plants were treated similarly but the bacterial suspension was replaced by sterile liquid medium XAS [59].

### 4.2. Leaf Tissue Sampling

Leaf samples were collected for transcriptome sequencing from the two cultivars at 0 (named R0_CK, and S0_CK), 24 (named R24_Xa, and S24_Xa), 48 (named R48_Xa, and S48_Xa), and 72 (named R72_Xa, and S72_Xa) hpi. At each sampling time point, inoculated leaf tissue (about 5 cm) was collected from six plants of each treatment and the leaf pieces were divided into three aliquots for further processing. These leaf samples were immediately snap-frozen in liquid nitrogen and stored at −80 °C until DNA and RNA extraction. Thus, a total of 24 samples were used for Illumina RNA-seq deep sequencing. Additionally, leaf scald symptoms (white short pencil lines) were recorded prior to each leaf sampling.

### 4.3. Quantification of Bacterial Populations in Inoculated Plants

Total genomic DNA was extracted from sugarcane leaf samples using the standard CTAB protocol [58]. The population size of *X. albilineans* in inoculated leaf samples was determined using a real-time TaqMan-based qPCR method [60]. For this purpose, plasmid DNA pMD-albI (3223 bp) was constructed by PCR amplification of the 529-nt *alb*I gene of *X. albilineans* with newly designed primer pair Xa-albI-F (5′-ACAAGGGCAGTTCCGCAATCCGCGT-3′) and Xa-albI-R (5′-GCAGCCGTAGTTGTTCCATAGC-3′), and subsequent cloning into vector pMD19-T (Takara, Daliang, China). The qPCR assay was performed in a final volume of 20 µL containing 1 µL DNA, 12.5 µL of TaqMan universal master mix (Roche Molecular Systems, Mannheim, Germany), 0.4 µL of each forward and reverse primer (10 µmol/L), and 0.625 µL of TaqMan double-quenched probe XaQ (2 µmol/L; Integrated DNA Technologies, Shanghai, China), and distilled water to the final volume. The qPCR optimum cycling conditions were as follows: 2 min at 50 °C, 10 min at 95 °C, 40 cycles of 15 s at 95 °C, and 1 min at 60 °C. Negative and blank controls consisted of total DNA (100 ng/µL) of a pathogen-free leaf of sugarcane and sterile distilled water, respectively. Three biological replicates and three technical replicates were used for all treatments.

An Applied Biosystems 7500 thermal Cycler (Alameda, CA, USA) was used to perform real-time qPCR and data analysis. When Ct value was less than 35, a sample was considered *X. albilineans* positive, whereas a sample with a Ct value exceeding 35 was considered negative. The DNA copy number of each sample was determined with Microsoft^®^ Excel by interpolation of the mean Ct value, which is defined by the crossing cycle number or crossing point, against the logarithm of the concentration of 10-fold dilution series of the pMD-albI standard curve equation. 

### 4.4. Library Construction and Illumina RNA-Sequencing

Total RNA was extracted using the TRIzol^®^ kit (Invitrogen, Carlsbad, CA, USA), following the manufacturer’s instructions. Extracted DNA or RNA was dissolved in nuclease-free water and their quality and integrity were measured by the Agilent Bio-analyzer 2100 system (Agilent Technologies, Santa Clara, CA, USA) and 1% agarose gel electrophoresis, respectively. The RNA concentration was estimated using a Qubit^®^ RNA Assay kit and the Qubit^®^ 2.0 Fluorometer (Life Technologies, CA, USA). Following RNA isolation and quality assessment, cDNA libraries were constructed and remaining nucleic acids was used for transcript analysis described below. An equal amount (1.5 µg) of total RNA was pooled from three biological replicates) from each sample for each cDNA library construction using the NEBNext^®^ Ultra^TM^ RNA Library Prep Kit for Illumina (NEB, MA, USA), following the manufacturer’s instructions. The libraries were sequenced using an Illumina NovaSeq 6000 platform to generate 150 bp paired-end reads (PE 150) were generated at Novogene Bioinformatics Institute, Beijing, China. The 24 libraries were named R0_CK (leaves of resistant cultivar LCP 85-384 inoculated with sterile liquid medium), R24_Xa, R48_Xa and R72_Xa (leaves of resistant cultivar LCP 85-384 inoculated with *X. albilineans* strain Xa-FJ1), S0_CK (leaves of susceptible cultivar ROC20 inoculated with sterile liquid medium), and S24_Xa, S48_Xa, and S72_Xa (leaves of susceptible cultivar ROC20 inoculated with *X. albilineans* strain Xa-FJ1). The Illumina sequencing data of sugarcane infected with *X. albilineans* were deposited into the United States National Center for Biotechnology Information (NCBI) SRA database under accession number PRJNA549590.

### 4.5. De Novo Transcriptome Assembly

Prior to performing bioinformatic analyses, clean reads (clean data) were obtained from the raw reads using in-house perl scripts. Low quality reads and reads containing adapters or poly-N were removed. At the same time, GC content, Q30, and sequence duplication levels of the clean data were determined. These clean data of high quality were then used to carry out all downstream analyses. One large left.fq file consisted of the pooled left files (read1 files) from all libraries/samples. The large right.fq file was formed by all the pooled right files (read2 files). Transcriptome assembly was performed using Trinity (v2012-10-05, Broad Institute, Cambridge, MA, USA) software based on the left.fq and right.fq files using the short read assembling program without a reference genome. The min kmer cov was set to 2 and all other parameters were set to default. The base calling and base quality assignments were assessed using PHRED [61].

### 4.6. Differential Gene Expression Analysis and Function Annotation

For each leaf sample, gene expression levels were estimated by RSEM (rsem-12.0) [62]. Clean data were initially mapped back onto the assembled transcriptome, and the read count for each gene was then generated from the mapping results among the two sugarcane cultivars and the treatments at 0, 24, 48, and 72 hpi. Before differential gene expression analysis, the read counts for each library were adjusted using the edger program package with a one-scale normalizing factor. DESeq2 (http://www.bioconductor.org/packages/release/bioc/html/DESeq2.html) was used for differential expression analysis of the two groups (three biological replications per group). This provided statistical methods for determining differential expression in the digital gene expression data, using a model based on the negative binomial distribution [63]. The resulting *p*-values were adjusted using the Benjamini and Hochberg approach for dealing with the false discovery rate [64]. Genes with an adjusted *p*-value < 0.005 (|log_2_(fold change)| ≥ 1) found by DESeq were considered as DEGs.

The following databases were used for gene annotation: NCBI non-redundant protein sequences (Nr at https://www.ncbi.nlm.nih.gov/), NCBI non-redundant nucleotide sequences (Nt at https://www.ncbi.nlm.nih.gov/), Protein family (Pfam at http://pfam.sanger.ac.uk/), Clusters of Orthologous Groups of proteins (KOG/COG at http://www.ncbi.nlm.nih.gov/COG/), a manually annotated and reviewed protein sequence databse (Swiss-Prot at http://www.ebi.ac.uk/uniprot/), KEGG Ortholog database (KO), and GO [65,66,67]. These databases were searched by BLASTx with the significant threshold E-value of 10^−5^. GO enrichment analysis of the DEGs was performed using GOseq R packages with Wallenium non-central hyper-geometric distribution, which can adjust for gene length bias in differentially expressed genes [68]. GO terms for each of the unigenes were based on the best BLASTx hit from the Nr and Pfam databases using Blast2GO software (version 2.5, BioBam Bioinformatics S. L., Valencia, Spain) with an E-value threshold of 10^−5^. Additionally, these annotated DEGs were further subjected to KEGG pathway enrichment analysis [66]. KOBAS v.2.0 (Center for Bioinformatics, Peking University, Beijing, China) was used to test statistical enrichment of differential expression genes in KEGG pathways [67,69].

### 4.7. Quantitative Real Time RT-PCR Analysis

To validate the expression pattern of DEGs identified by the RNA-seq analysis, ten pairs of specific primers for selected DEGs and one primer pair for reference gene *GAPDH* (Table S4) were designed using Beacon Designer software version 8.20 (Premier Biosoft International, Palo Alto, CA, USA). Transcript expression levels of these genes were determined by qRT-PCR at each time point. The qPCR was performed using TB Green^TM^ Premix Ex Tag^TM^ II (Tli RNaseH Plus) (Takara, Dalian, China) on the QuantStudio^®^ Real-Time PCR system (Applied Biosystems, Foster City, USA). Each reaction was performed in a final volume of 20 µL containing 10 µL of TB Green^TM^ Premix Ex TaqTM (Tli RNaseH Plus), 0.4 µL of 10 mM forward primer, 0.4 µL of 10 mM Reverse primer, 2.0 µL of the cDNA sample, 0.4 µL of ROX Reference Dye II, and distilled water to the final volume. The qPCR cycling was performed at 50 °C for 2 min, 15 min polymerase activation at 95 °C, 40 cycles at 95 °C for 15 s and 60 °C for 34 s, and finally a dissociation stage (melt curve) at 95 °C for 15 s, 60 °C for 1 min, and 95 °C for 15 s. A melting curve analysis was conducted to confirm qPCR specificity. The Glyceraldehyde 3-phoshate dehydrogenase (*GAPDH*) gene was used as a house keeping gene to normalize the amount of template cDNA added in each reaction. To determine the relative fold differences for each sample, the Ct value for each gene was normalized to the Ct value of the reference gene. The qRT-PCR results were analyzed by the 2^-ΔΔCt^ quantitative method to determine the differences in gene expression [70]. qRT-PCR analysis was carried out with three biological and three technical replicates.

### 4.8. Statistical Analyses

qRT-PCR and RNA-seq data at the same time-point were compared using the paired comparison T-test in SAS version 8.1 (SAS Institute, Cary, NC, USA). Additionally, a general linear model was fitted to all qRT-PCR datasets (Relative expression level of different time-points for each cultivar) using the one-way ANOVA procedure. Multiple comparisons of the means were conducted by the Student–Newman–Keuls (SNK) Test.

## 5. Conclusions

This study provides the first transcriptome dataset of 24 cDNA libraries of sugarcane in response to infection by *X. albilineans*, the causal agent of leaf scald. A large number of DEGs and their corresponding pathways were identified in both cultivars, such as plant–pathogen interaction, glutathione metabolism, and plant hormone signal transduction pathways. Based on both RNA-seq and qRT-PCR data, genes related to auxin and ethylene signal transduction pathways were differentially expressed in the two cultivars after *X. albilineans* infection*,* suggesting that these genes play key roles in sugarcane during infection by *X. albilineans.* Transcriptome analysis of additional cultivars differing in resistance of leaf scald, as well as functional gene analyses, need to be performed to validate these findings.

## Figures and Tables

**Table 1:** Length distribution of assembled transcripts and unigenes.

Characteristics of Transcripts/Unigenes	Number of Transcripts (451,856,714 Nucleotides)	Number of Unigenes (429,371,352 Nucleotides)
200–500 bp length	315,474 (51%)	239,843 (45%)
500 bp-1 kbp length	164,820 (27%)	162,182 (30%)
1–2 kbp length	100,096 (16%)	99,766 (19%)
˃2 kbp	33,880 (5.5%)	33,864 (6%)
Total	614,270 (100%)	535,655 (100%)
Minimum length (bp)	201	201
Average length (bp)	736	802
Median length (bp)	487	555
Maximum length (bp)	17,717	17,717
N50 (bp) ^a^	1045	1102
N90 (bp) ^b^	324	369

^a^ The N50 value is the contig length for which 50% of the assembly corresponds to contigs of this size or larger. ^b^ The N90 value is the contig length for which 90% of the assembly has contigs of this size or larger.

**Table 2:** Transcriptional level of ten selected candidate genes involved in plant hormone signal transduction pathways (ko047075) based on RNA-sequencing data in sugarcane cultivars infected by *X. albilineans* ^a^.

Gene ID	KEGG Orthology	Gene description	LCP 85-384			ROC20			
			24 hpi	48 hpi	72 hpi	24 hpi	48 hpi	72 hpi	
Cluster-4871.238958	K14484	Auxin-responsive protein IAA (IAA)	1.10	1.19	1.07	2.57	3.66	2.83
Cluster-4871.233479	K14487	Auxin responsive GH3 gene family (GH3)	−1.26	−0.63	−0.61	0.08	−0.04	0.26
Cluster-4871.390584	K14488	SAUR family protein (SAUR)	4.69	2.90	3.66	−0.42	−1.63	0.73
Cluster-4871.255133	K12126	Phytochrome-interacting factor 3 (PIF3)	−0.34	−0.45	−1.03	2.27	2.27	1.65
Cluster-4871.248068	k14496	Abscisic acid receptor PYR/PYL family (PYL)	1.49	1.30	1.47	0.90	0.74	0.69
Cluster-4871.245620	K14498	Serine/threonine-protein kinase SRK2 (SNRK2)	1.49	1.30	1.47	0.90	0.74	0.69
Cluster-4871.356618	K14510	Serine/threonine-protein kinase CTR1 (CTR1)	2.51	2.05	1.85	1.39	0.45	0.31
Cluster-4871.244221	K14513	Ethylene-insensitive protein 2 (EIN2)	−0.69	−0.46	−0.46	−1.16	−1.04	−1.24
Cluster-4871.211143	K14506	Jasmonic acid-amino synthetase (JAR1)	−0.27	−0.03	0.38	−1.62	−1.73	−1.90
Cluster-4871.226184	K14508	Regulatory protein NPR1 (NPR1)	2.92	3.69	4.46	−0.91	−0.59	−0.55

^a^ Relative gene expression levels are presented as log_2_ (fold change). KEGG, Kyoto Encyclopedia of Genes and Genomes. hpi, hours post-inoculation.

## Supplementary Materials

Supplementary materials can be found at https://www.mdpi.com/1422-0067/20/24/6138/s1.
