# Supplementary material for: Comparative Transcriptome Profiling of Resistant and Susceptible Sugarcane Cultivars in Response to Infection by Xanthomonas albilineans
Source: Int J Mol Sci. 2019 Dec 5;20(24):6138. doi: 10.3390/ijms20246138 (PMC6940782; doi:10.3390/ijms20246138)
Supplement: Supplementary file 1 [file ijms-20-06138-s001.zip › Supplementary files-20191205/Figure S1-20191031.pdf]

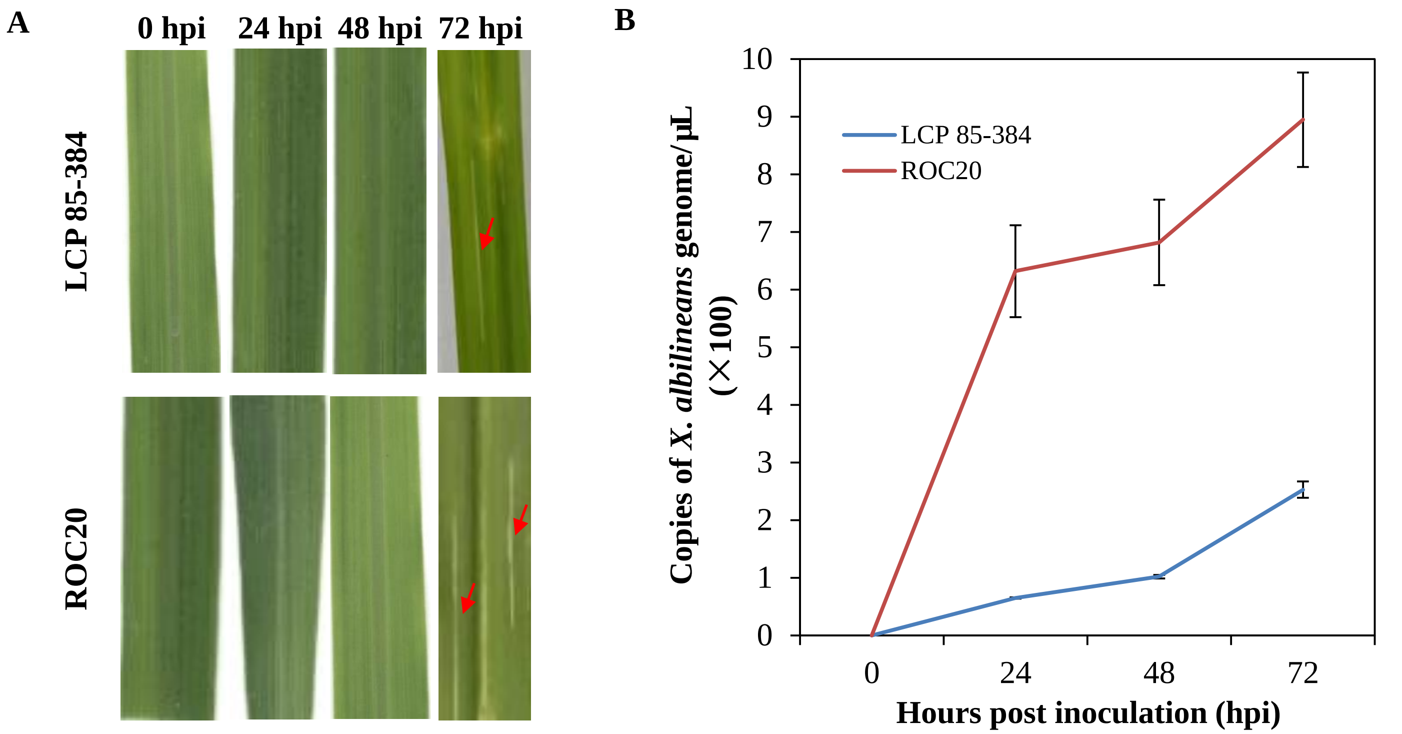

Figure S1 Leaf scald symptoms observed in the resistant (LCP 85-384) and susceptible (ROC20) sugarcane cultivars after inoculation with *X. albilineans* (strain Xa-FJ1). (A) Pencil line streak (red arrow); (B) Quantification of *X. albilineans* populations in sugarcane cultivars using a real-time quantitative PCR (qPCR) assay. Inoculated plants collected at each time-point were distributed into three equal amounts of leaves for RNA-seq and qPCR. 0 hpi = mock (inoculated with sterile medium). Data at each time-point correspond to three biological replicates and three technical replicates.
