# Supplementary material for: Comparative Transcriptome Profiling of Resistant and Susceptible Sugarcane Cultivars in Response to Infection by Xanthomonas albilineans
Source: Int J Mol Sci. 2019 Dec 5;20(24):6138. doi: 10.3390/ijms20246138 (PMC6940782; doi:10.3390/ijms20246138)
Supplement: Supplementary file 1 [file ijms-20-06138-s001.zip › Supplementary files-20191205/Figure S2-20191031.pdf]

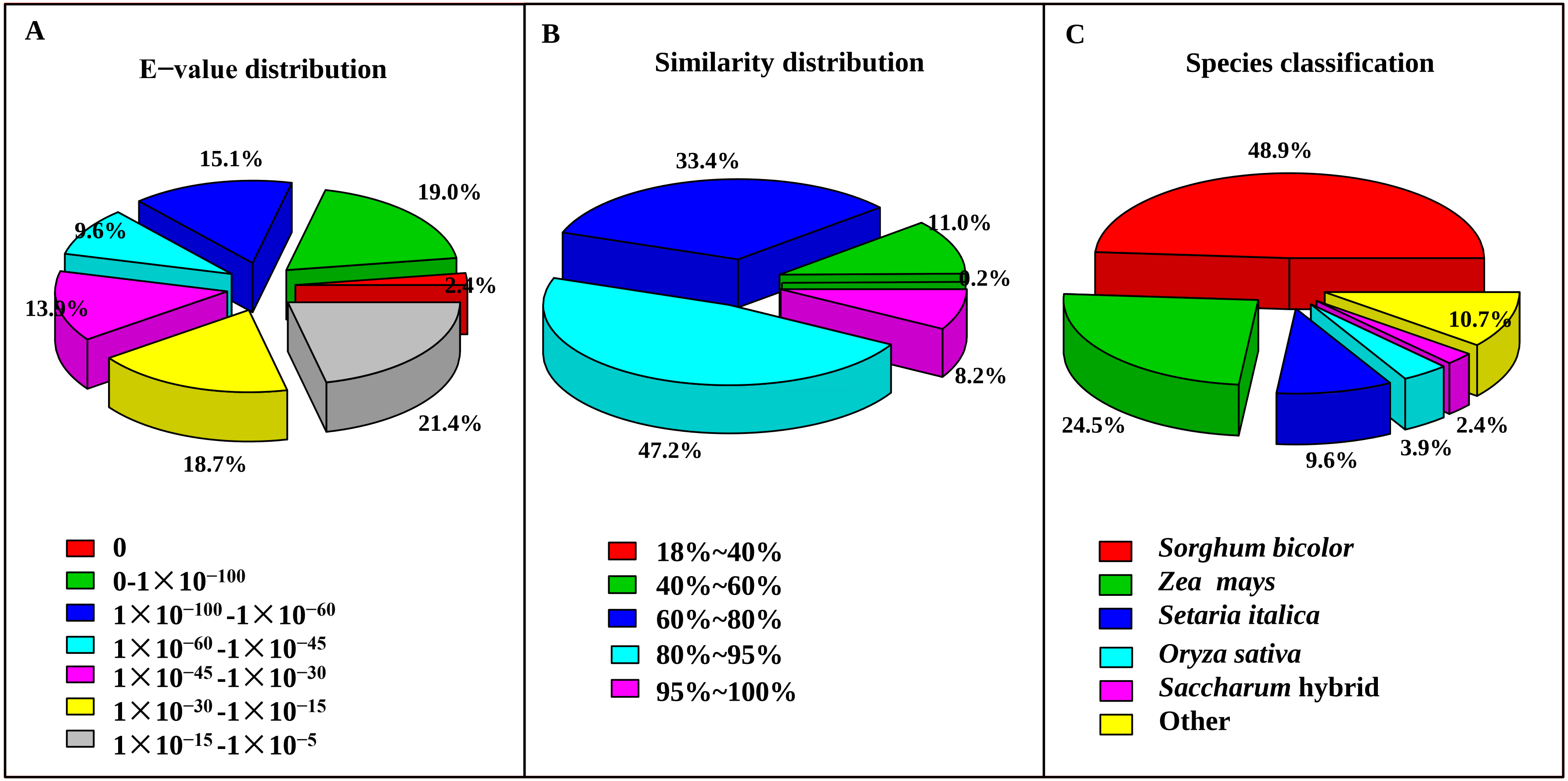

Figure S2 Comparative analysis of sugarcane transcriptomic sequences with known Nr database sequences including *Sorghum bicolor*, *Zea mays*, *Setaria italica*, *Oryza sativa*, *Saccharum* hybrid spp., and other crops. (A) E-value distribution, (B) Similarity distribution with the Nr database, (C) Species classification of annotated unigenes.
