# Supplementary material for: Comparative Transcriptome Profiling of Resistant and Susceptible Sugarcane Cultivars in Response to Infection by Xanthomonas albilineans
Source: Int J Mol Sci. 2019 Dec 5;20(24):6138. doi: 10.3390/ijms20246138 (PMC6940782; doi:10.3390/ijms20246138)
Supplement: Supplementary file 1 [file ijms-20-06138-s001.zip › Supplementary files-20191205/Figure S3-20191031.pdf]

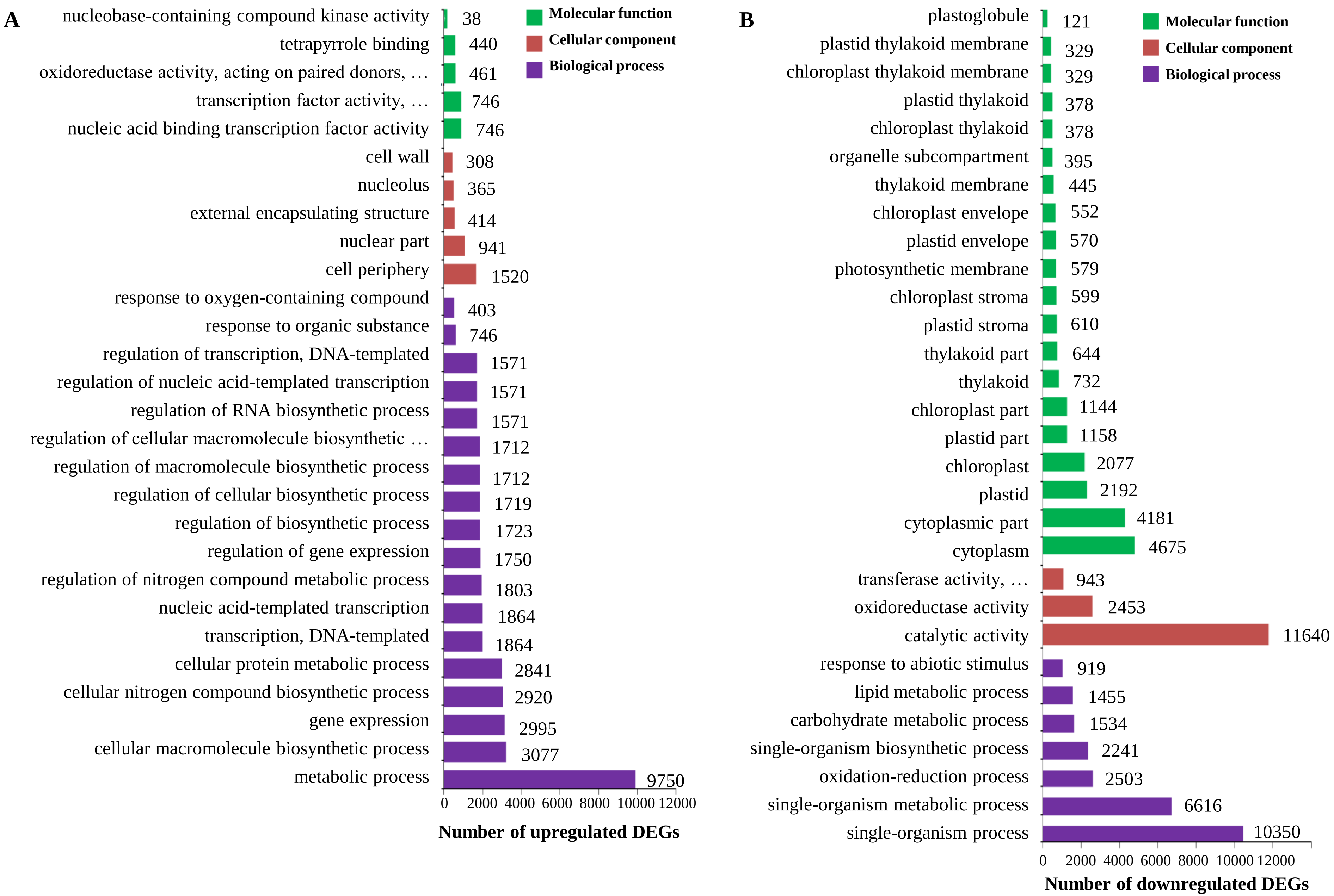

Figure S3. Assignment to Gene Ontology (GO) categories of differentially expressed genes (DEGs) of susceptible sugarcane cultivar ROC20 infected by *X. albilineans*. (A) Upregulated DEGs, (B) Downregulated DEGs.
