# Supplementary material for: Comparative Transcriptome Profiling of Resistant and Susceptible Sugarcane Cultivars in Response to Infection by Xanthomonas albilineans
Source: Int J Mol Sci. 2019 Dec 5;20(24):6138. doi: 10.3390/ijms20246138 (PMC6940782; doi:10.3390/ijms20246138)
Supplement: Supplementary file 1 [file ijms-20-06138-s001.zip › Supplementary files-20191205/Figure S4-20191205.pdf]

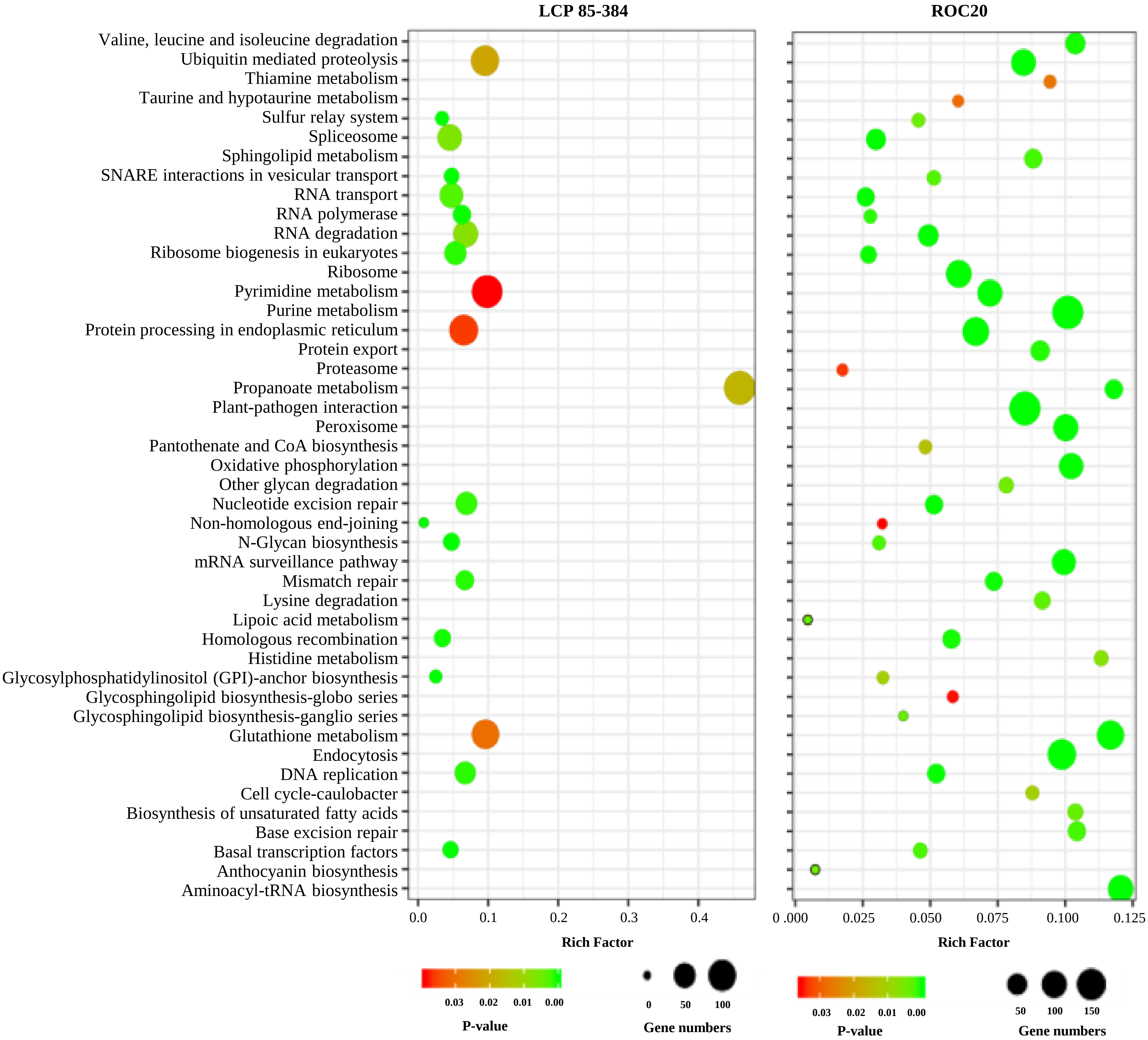

Figure S4. KEGG pathway classification and functional enrichment of downregulated DEGs (statistically significant ≥2-fold, P-value < 0.005) in LCP 83-384 (left panel) and ROC20 (right panel). The X-axis represents the enrichment factor (Rich Factor) which is the ratio of the foreground value (the number of DEGs) and the background value (total gene amount). The Y-axis shows the pathway names. A larger value of the Rich Factor indicates a higher enrichment value. The color indicates the P-value (high: red, low: green), A lower P-value refers to a more significant enrichment. Point size indicates DEG number and larger dots refer to higher numbers of DEGs.
