# Supplementary material for: Comparative Transcriptome Profiling of Resistant and Susceptible Sugarcane Cultivars in Response to Infection by Xanthomonas albilineans
Source: Int J Mol Sci. 2019 Dec 5;20(24):6138. doi: 10.3390/ijms20246138 (PMC6940782; doi:10.3390/ijms20246138)
Supplement: Supplementary file 1 [file ijms-20-06138-s001.zip › Supplementary files-20191205/Figure S5-20191031.pdf]

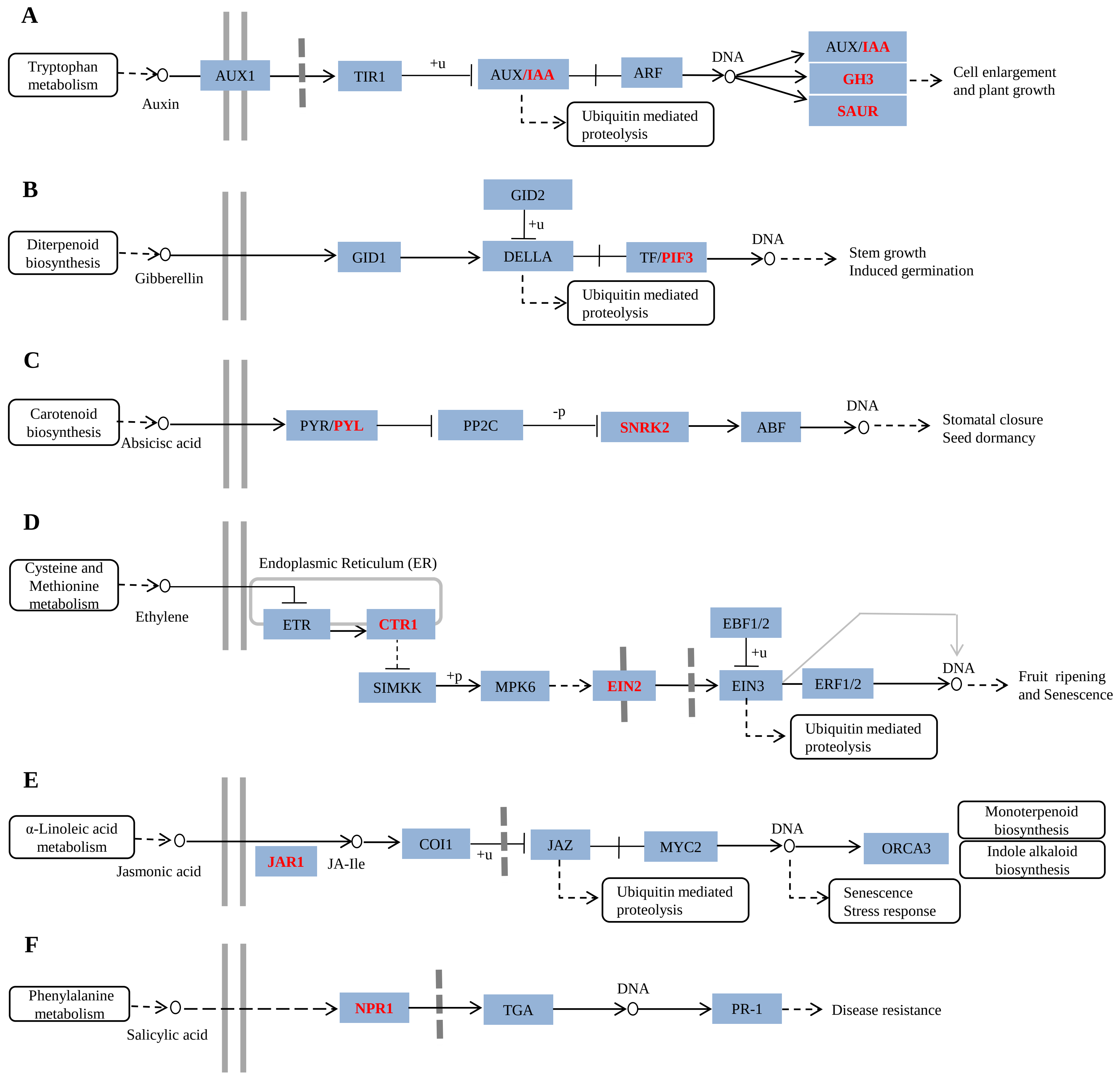

Figure S5 Ten DEGs involved in plant hormone signal transduction pathways. The sketches of these pathways were automatically made by KEGG online (http://www.genome.jp/kegg/). Two solid pillars present the cytomembrane and dotted pillar presents the nuclear membrane; o, chemical molecules; +p, phosphorylation; -p, dephosphorylation; +u, ubiquitylation; -u, ubiquitylation. Selected genes that were used for validation by qRT-PCR are written in red bold.
