# Supplementary material for: Comparative Transcriptome Profiling of Resistant and Susceptible Sugarcane Cultivars in Response to Infection by Xanthomonas albilineans
Source: Int J Mol Sci. 2019 Dec 5;20(24):6138. doi: 10.3390/ijms20246138 (PMC6940782; doi:10.3390/ijms20246138)
Supplement: Supplementary file 1 [file ijms-20-06138-s001.zip › Supplementary files-20191205/Table S1-20191031.doc]

Table S1 Summary of the read numbers and alignment from RNA sequencing ^a^

|  | Leaf scald resistant cultivar (LCP 85-384) | | | | Leaf scald susceptible cultivar (ROC20) | | | |
| --- | --- | --- | --- | --- | --- | --- | --- | --- |
| Description | R0_CK | R24_Xa | R48_Xa | R72_Xa | S0_CK | S24_Xa | S48_Xa | S72_Xa |
| Raw reads | 62,175,579 | 58,882,140 | 58,033,567 | 57,563,635 | 57,924,462 | 51,506,253 | 52,919,223 | 62,100,971 |
| Clean reads | 60,577,163 | 56,984,407 | 56,581,930 | 55,908,242 | 56,171,055 | 50,221,927 | 51,509,165 | 60,390,747 |
| Total mapped | 40,197,131 | 37,111,831 | 36,343,853 | 35,824,660 | 37,597,507 | 32,757,493 | 34,095,181 | 38,473,521 |
| Mapped % | 66.4 | 65.1 | 64.2 | 64.1 | 66.9 | 65.2 | 66.2 | 63.7 |
| Clean bases (Gb) | 9.09 | 8.55 | 8.49 | 8.39 | 8.43 | 7.53 | 7.73 | 9.06 |
| Error rate（%） | 0.02 | 0.02 | 0.02 | 0.02 | 0.02 | 0.02 | 0.02 | 0.02 |
| Q30 (%) | 90.8 | 90.7 | 90.2 | 91.7 | 91.0 | 92.0 | 91.9 | 91.8 |
| GC content (%) | 57.1 | 56.6 | 56.1 | 57.0 | 58.3 | 57.8 | 57.1 | 58.3 |

^a^ Leaves of two sugarcane cultivars with different resistance level to *X. albilineans* (LCP 85-384, resistant, ROC20, susceptible) were sampled for RNA sequencing at different time periods after inoculation with the pathogen: 0 (control), 24h (Xa), 48h (Xa), and 72 h (Xa) hours post inoculation. Three replicates were set for each treatment. Raw reads: the numbers of original sequence data, Clean reads: total number of reads after filtering low-quality reads; Total mapped: total number of reads that can be mapped to the reference genome, Clean bases: *Qphred* = -10*log10(e)*, Error rate (%): sequencing error rate; Q30(%): percentage of bases with a Phred value ˃ 30, GC content: the percentage of G and C bases in all clean reads.
