# Supplementary material for: Comparative Transcriptome Profiling of Resistant and Susceptible Sugarcane Cultivars in Response to Infection by Xanthomonas albilineans
Source: Int J Mol Sci. 2019 Dec 5;20(24):6138. doi: 10.3390/ijms20246138 (PMC6940782; doi:10.3390/ijms20246138)
Supplement: Supplementary file 1 [file ijms-20-06138-s001.zip › Supplementary files-20191205/Table S2-20191031.doc]

Table S2 Summary of the gene annotation

| Database ^a^ | Number of unigenes  (Total = 535,655) | Percent of unigenes present in the database |
| --- | --- | --- |
| Nr | 263,129 | 49 |
| Nt | 356,538 | 67 |
| KO | 71,456 | 13 |
| Swiss-Prot | 147,109 | 27 |
| Pfam | 172,132 | 32 |
| GO | 199,233 | 37 |
| KOG | 42,942 | 8 |
| All databases | 23,075 | 4 |
| At least one database | 407,743 | 76 |

^a^ All the assembled high-quality unigenes were annotated after BLASTx search using seven public databases: NCBI non-redundant protein (Nr), NCBI nucleotide sequences (Nt), Kyoto Encyclopedia of Genes and Genomes (KEGG) Ortholog (KO), manually annotated and reviewed sequences (Swiss-Prot), Protein family (Pfam), Gene Ontology (GO), eukaryotic Ortholog Groups (KOG).
