# Supplementary material for: Comparative Transcriptome Profiling of Resistant and Susceptible Sugarcane Cultivars in Response to Infection by Xanthomonas albilineans
Source: Int J Mol Sci. 2019 Dec 5;20(24):6138. doi: 10.3390/ijms20246138 (PMC6940782; doi:10.3390/ijms20246138)
Supplement: Supplementary file 1 [file ijms-20-06138-s001.zip › Supplementary files-20191205/Table S4-20191031.doc]

Table S4 Primers used for the quantitative reverse transcription-PCR (qRT-PCR) assay of ten differentially expressed genes (DEGs) and the reference gene in sugarcane.

| Target gene ^a^ | Forward primer (5'-3') | Reverse primer (5'-3') | Tm (^o^C) ^b^ | Amplicon size (bp) | Efficiency ^c^ |
| --- | --- | --- | --- | --- | --- |
| *IAA* | TGGTGAATACACTTTAATTTGTG | GCAGAAACAAATACATTCCATG | 63 | 84 | 0.98 |
| *GH3* | GATCGTCTCCACCGCGTACTTCTCCTC | CACCTCGTCCGTCTTGTCCGACTCTAC | 66 | 346 | 1.00 |
| *SAUR* | TCATCGTCACTCGCCCAAGCTCCCA | TGCCGTTCTTCCTCTTCCACGCCATG | 65 | 118 | 1.00 |
| *PIF3* | GGACTCCTTTTCAAGTGATAGATC | GGAAGAAATGAAGCTGAAACATA | 64 | 152 | 0.99 |
| *PYL* | GACGAATCAATCAATCAATCAA | TTGCGATTGTATTATTATAGCAG | 60 | 144 | 0.99 |
| *SNRK* | CCATCCATGGTTTCTAAAGAAC | TAGCCTGATCTCAATGATTTTG | 54 | 163 | 0.99 |
| *CTR1* | GAGGATACTCAATCTGGACTAA | TGCTCCATTAGAATCTTACTG | 65 | 179 | 0.98 |
| *EIN2* | CCAACTATATGTTTAGTGGTTC | GAAGGTTTATACCAAATAAATGC | 54 | 106 | 0.99 |
| *JAR1* | GGAAGATCTATGTGCTGATATAAG | CCCATACCAATTACTCAAATTC | 65 | 154 | 1.02 |
| *NPR1* | GCTTCTTAACTTTGTAGACAAG | CTAGCAATCCTTTGAATACATTTG | 55 | 144 | 0.97 |
| *GAPDH* | CACGGCCACTGGAAGCA | TCCTCAGGGTTCCTGATGCC | 58 | 101 | 1.01 |

^a^ *GAPDH* was used as a reference gene.

^b^ Tm = melting temperature of primers.

^c^ Efficiencies of SYBR green based qRT-PCR assays were calculated based on the serial five-time dilutions of total cDNA of the sugarcane leaf sample.
